# Design, rationale and analysis plan for the Stand Up for Health trial in contact centres: a stepped wedge feasibility study

**DOI:** 10.1186/s40814-020-00683-1

**Published:** 2020-09-23

**Authors:** Richard A. Parker, Jillian Manner, Divya Sivaramakrishnan, Graham Baker, Andrew Stoddart, Scott Lloyd, Ruth Jepson

**Affiliations:** 1 Edinburgh Clinical Trials Unit, Usher Institute, University of Edinburgh, Edinburgh, UK; 2 Scottish Collaboration for Public Health Research and Policy (SCPHRP), School of Health in Social Science, University of Edinburgh, Edinburgh, UK; 3 Physical Activity for Health Research Centre, Institute for Sport, P.E. and Health Sciences, Moray House School of Education and Sport, University of Edinburgh, Edinburgh, UK; 4 Public Health South Tees, The Live Well Centre, Dundas House, Dundas Arcade, Middlesbrough, UK; 5 Fuse – UKCRC Centre for Translational Research in Public Health, Population Health Sciences Institute, William Leech Building, Newcastle University, Newcastle upon Tyne, UK; 6 School of Health and Social Care, Teesside University, Centuria Building, Middlesbrough, UK

## Abstract

**Background:**

Contact centres are one of the most sedentary workplaces, with employees spending a very high proportion of their working day sitting down. About a quarter of contact centre staff regularly experience musculoskeletal health problems due to high levels of sedentary behaviour, including lower back pain. There have been no previous randomised studies specifically aiming to reduce sedentary behaviour in contact centre staff. To address this gap, the Stand Up for Health (SUH) study aims to test the feasibility and acceptability of a complex theory-based intervention to reduce sedentary behaviour in contact centres.

**Methods:**

The Stand Up for Health study has a stepped wedge cluster randomised trial design, which is a pragmatic design whereby clusters (contact centres) are randomised to time points at which they will begin to receive the intervention. All contact centre staff have the opportunity to experience the intervention. To minimise the resource burden in this feasibility study, data collection is not continuous, but undertaken on a selective number of occasions, so the stepped wedge design is “incomplete”. Eleven contact centres in England and Scotland have been recruited, and the sample size is approximately 27 per centre (270 in total). The statistical analysis will predominantly focus on assessing feasibility, including the calculation of recruitment rates and rates of attrition. Exploratory analysis will be performed to compare objectively measured sedentary time in the workplace (measured using an activPAL™ device) between intervention and control conditions using a linear mixed effects regression model.

**Discussion:**

To our knowledge, this is the first stepped wedge feasibility study conducted in call centres. The rationale and justification of our novel staircase stepped wedge design has been presented, and we hope that by presenting our study design and statistical analysis plan, it will contribute to the literature on stepped wedge trials, and in particular feasibility stepped wedge trials. The findings of the study will also help inform whether this is a suitable design for other settings where data collection is challenging.

**Trial registration:**

The trial has been registered on the ISRCTN database: http://www.isrctn.com/ISRCTN11580369

## Background

Sedentary behaviour is known to contribute to poor health outcomes such as poor mental health, musculoskeletal disorders, diabetes and cardiovascular disease [1–6]. These risks are independent of physical activity [7, 8]. Conceptually, sedentary behaviour and physical activity are different [9], with each thought to pose health risks independent of each other [5, 10–12]. Recent studies have concluded that physical activity modifies the association between sedentary behaviour and cardiovascular disease, cancer, and all-cause mortality [13–15]. Additionally, in populations with lower physical activity, sedentary behaviour is associated with greater health risks [13–15]. As evidenced by a recent review [16], interventions which showed the most promise in reducing sitting time were those that aimed to directly change sedentary behaviour rather than indirectly through increasing physical activity. The reduction of sedentary behaviour is therefore not a consequence of effectively promoting physical activity and should be recognised independently when developing interventions, guidelines and legislation.

Many employees working in office-based environments become exposed to prolonged periods of inactivity in static seated postures, which are enforced by factors such as ergonomic set-up and workplace culture [17]. This sedentary behaviour can impact significantly on the daily lives and activities of workers. For example, musculoskeletal issues are one of the most prevalent occupational health problems for desk-based workers and are a leading cause for disability worldwide [18–20]. Multi-component interventions have previously been successful in reducing total [21] and prolonged [22] sedentary behaviour within this setting. However, few studies have been effective at promoting physical activity [23] in the workplace and only a limited number of trials have reported success in managing long-term positive sedentary behaviour change [22]. In addition to other study and population characteristics, the heterogeneity of workplaces involved in some of these studies is likely to have contributed to mixed findings.

Contact centres (also known as call centres) are associated with higher levels of sedentary behaviour than other office-based workplaces [4, 24, 25]. Employees typically spend up to 90% of their working day sitting down [24]. The technology used by staff in contact centres prevents them from regularly leaving their desk and many call handlers often report stressful work environments due to low workplace autonomy, strict supervision of individual performance and commission-based salary systems [26]. Contact centre staff may therefore be of greater risk of developing non-communicable diseases [24]. Additionally, one in four members of contact centre staff regularly experience musculoskeletal problems with 22.4% of sick days lost to such problems [27]. At present, only a very limited number of research studies have sought to explore the reduction of sedentary behaviour or promotion of physical activity in the contact centre setting.

The combination of high levels of sedentary behaviour, and the number of constraining factors, suggest that call centres require tailored interventions. However, there have been few attempts to do so [28–31]. Feasibility work with centres in Liverpool, UK, identified a number of challenges to reducing sedentary behaviour and incorporating physical activity into the shifts of call centre workers [29]. These included having sufficient buy-in from team leaders and wider stakeholders to support intervention activities to reduce sedentary behaviour, and high call centre staff attrition. This is why further research into developing sustainable and scalable interventions in contact centres is vitally important; to ensure healthier working policies are distributed equitably across all workplaces, not just those that have more worker autonomy and better workplace conditions.

To date, the evaluations of interventions to reduce sedentary behaviour and increase physical activity conducted in contact centres have all been non-randomised, quasi-experimental studies and most were small pilot or feasibility studies with short study durations. Only one of the studies was conducted within the UK, but this study was only conducted in a single contact centre with very small sample size (13 individuals completing follow-up), and with a short study duration of 8 weeks [29]. Randomised designs are needed because they are less susceptible to biases, but this has to be balanced by what is pragmatic in this setting. Individually randomised designs (i.e., randomisation occurs within one contact centre/workplace) are unlikely to be viable because of the likelihood for contamination between intervention and control participants. In contrast, cluster randomised designs are more feasible because interventions are applied at the centre level.

A type of cluster randomised design called a stepped wedge trial involves randomising clusters to *time points* at which they will start to receive the intervention [32]. This design is particularly appealing for public health interventions since it has a number of features that make it more practical to deploy, such as the staggered implementation of the intervention and giving every site the opportunity to experience the intervention. This design has been used in practice since as far back as 1987, but was rarely seen in the literature until around 2007 [33]. Since then its popularity has increased greatly, especially since 2010 [32]. Although stepped wedge designs are now commonly seen in the literature, feasibility studies with a stepped wedge design are not. In a recent systematic review of feasibility studies conducted in preparation for future stepped wedge trials by Kristunas and colleagues, only three studies were identified that were themselves stepped wedge trials [34]. However, since that study has been published a non-systematic review of the literature by ourselves has identified at least seven new studies that have a stepped wedge feasibility study design [35–41] (albeit three of the studies appear to have come from the same research group). It therefore appears that this feasibility design has undergone a surge in popularity since 2018. However, our study includes some specific features of interest such as the way in which we collect data. The stepped wedge design of this feasibility study is recommended when preparing for a larger future stepped wedge trial [34, 42].

Stand Up for Health (SUH) is a multi-centre, feasibility study incorporating a stepped wedge trial design. It aims to test the feasibility and acceptability of a complex theory-based intervention to reduce sedentary behaviour in contact centres. The main aim of this paper is to present the rationale for the stepped wedge study design used in Stand Up for Health. This is important, not only because of the limited number of such study designs in the literature, but also because our design does not have the typical layout and measurement frequency seen in most other types of stepped wedge design, which requires justification. We also present our statistical and health economic analysis plan for analysing the data from the trial and assessing the feasibility of future trials.

## Methods

### Study aims

The primary aim of the study is to test the acceptability and feasibility of implementing the Stand Up for Health intervention in contact centres and assess the feasibility of using the stepped wedge cluster randomised controlled trial study design. As a secondary aim of the study, we also intend to scope the feasibility of a future health economic evaluation of Stand Up for Health.

### Intervention

Stand Up for Health (SUH) is an adaptive theory-based complex intervention to reduce sedentary time built around four theories of change: organisational, environmental, social/cultural, and individual. The focus is on fidelity to the theories of change rather than the specific activities (so an environmental change in one centre may be the use of standing desks, whilst in another it may be change in usage of space). The intervention will be delivered at the contact centre level with data collected at both the contact centre and individual levels. Individuals will be employees of the contact centres including managers, supervisors and call operators in Scotland and England (primarily the North of England). It is not the purpose of this paper to report on the intervention design or content in detail. Further details can be found on request to the corresponding author or here https://www.fundingawards.nihr.ac.uk/award/17/149/19.

### Comparator

For sites in which the intervention has not been introduced at the first time point (July 2019), they will start in the control condition before eventually receiving the intervention up to 12 months later. Whilst sites are under the control condition, no component of the Stand Up for Health intervention will be given.

### Reporting

The trial will be reported in a manner consistent with the stepped wedge extension [43] and the pilot study extension [44] to the Consolidated Standards of Reporting Trials (CONSORT) reporting guidelines.

### Study design

Stepped wedge trials are a type of cluster-randomised trial in which clusters are randomised to sequences rather than groups; and these sequences determine the specific time point at which clusters are supposed to cross over from the control to the intervention condition [43, 45, 46]. In standard cluster randomised trials, clusters in the control group are time-matched to intervention clusters and so no adjustment for natural (non-intervention related) changes over time is necessary. However, in stepped wedge cluster trials, time is a confounder by design, and therefore this factor needs to be taken into account in the analysis.

**Fig. 1:**
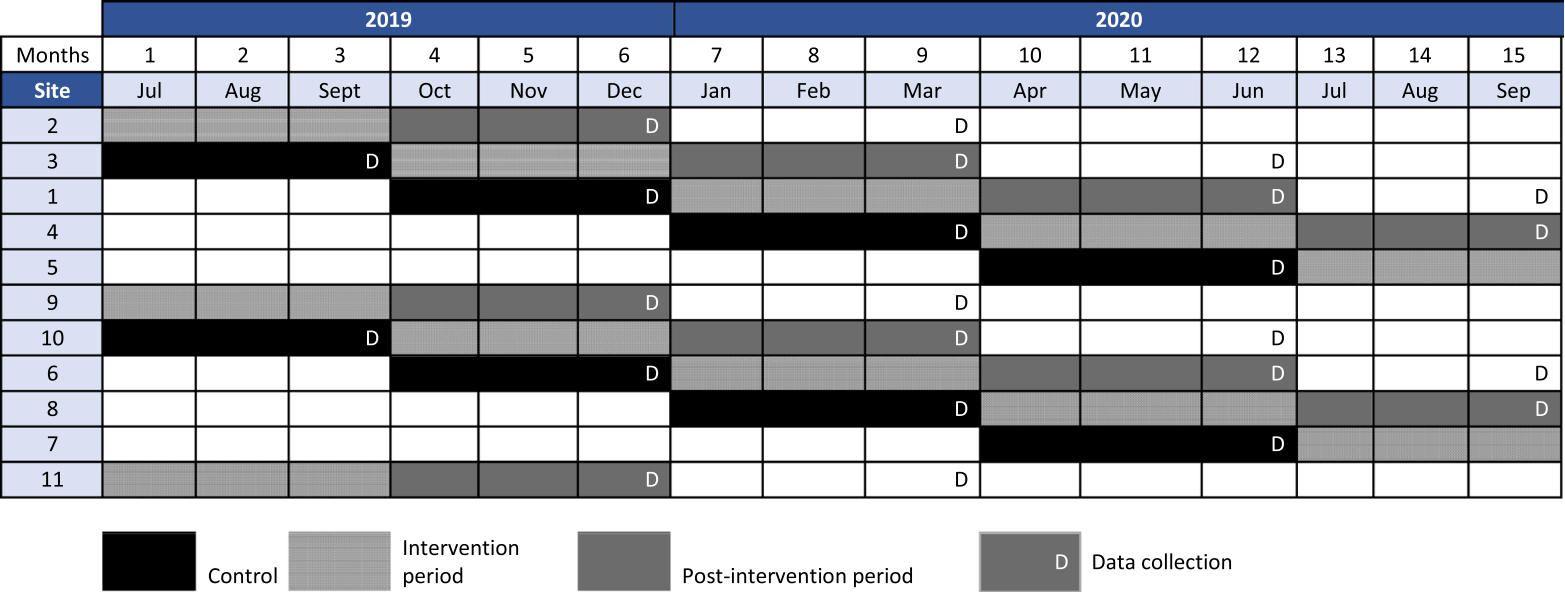
Schematic diagram of the stepped wedge intervention implementation and data collection

In the case of Stand Up for Health, the clusters are individual contact centres that are randomised to specific sequences or “rows” in the stepped wedge design (see Fig. 1). We randomised 11 contact centres to one of 5 unique sequences in Fig. 1, with each sequence corresponding to specific intervention start dates and data collection time points. There are three key 3-monthly periods (control, intervention and post-intervention), with data collection at the end of the control period, post-intervention period, and also 3 months after the end of the post-intervention period. There is a 3-monthly duration between each step in the stepped wedge diagram (Fig. 1).

However, unlike typical stepped wedge trial designs [46], the Stand Up for Health trial does not measure outcomes or recruit participants in every cluster-period (i.e. not in every possible time period defined by cluster or time), since this was deemed to create an unnecessarily high resource burden in this feasibility study. Accordingly, the trial could be described as an “incomplete cross-forward cluster randomised design” [47] because although all sites cross over from the control to the intervention condition, the stepped wedge design is “incomplete” because measurements are taken in only a proportion of all possible cluster-periods. Another name for this design is a “staircase” design, which has already been suggested as a potential highly efficient trial design by Kasza and Forbes, and broadly similar designs have already been adopted in practice [48–51]. The rationale for this specific stepped wedge design is that data collection is concentrated in those cluster periods that provide the most information about the treatment effect (i.e. close to the time the sites begin to receive the intervention) [48, 49].

It is also important to note that the study has an “open cohort” design as described by Copas et al. such that participants are repeatedly assessed over a series of measurement points and participants can join and leave the study throughout its duration [52]. Thus, we are expecting some differences in the group of participants assessed at the different data collection time points, although we expect that participants will remain mostly the same.

### Rationale

Individual-level randomisation was not possible as the SUH intervention was designed to be implemented at the level of the workplace/contact centres; hence, randomisation could only occur at the cluster level.

A stepped wedge trial design was chosen rather than standard parallel group cluster randomised design for several reasons:

Firstly, resource and cost limitations encountered during projects of this nature often make it infeasible to roll out the intervention and collect data simultaneously from all clusters at the same time as in a traditional parallel-group cluster design. The stepped wedge design offers an advantage to this by staggering the implementation of the intervention. This was of specific importance in the SUH project where it was proposed that 10 contact centres from a wide geographical spread (across Scotland and England) were recruited.

Secondly, stepped wedge trials are particularly suited for interventions that are unlikely to lead to harm, since in a stepped wedge design everyone receives the intervention [53]. In keeping with other interventions designed to increase sedentary behaviour, the Stand Up for Health intervention is unlikely to lead to harm. Indeed, it may even bring positive benefits to those individuals partaking in the intervention. The intervention has already been implemented in one contact centre for over a year and only positive benefits have been reported.

Thirdly, the intervention is such that it may be difficult to “unlearn” or completely abandon the principles learnt. Study designs with crossover from the intervention back to control are therefore not appropriate. In contrast, stepped wedge designs are ideal for these types of interventions [54].

Fourth, our experience is that contact centres are already wanting to implement the intervention when they hear about it, so it was important that all contact centres had an opportunity to ‘experience’ the intervention. Randomising contact centres to a control group without the opportunity to experience the intervention might have affected recruitment and contact centre engagement.

Fifth, despite the fact that contact centres share many common characteristics, there is also variability in terms of contact centre missions (e.g. sales, or services such as police and NHS); sector (private or public); size (from 20-2000); shift patterns; and demographics of the call centre staff. This between-site variability could result in differences in terms of uptake of the intervention, as well as in the outcome such as sedentary behaviour. We placed no exclusion criteria regarding the types of contact centres recruited. The majority of the centres are located in the Northeast of England, with one in London and two in Southern Scotland. Five are public sector, including emergency services and a community council. The remaining centres include banks, an insurance company and a telecommunications company. Shift patterns vary; some centres employ staff to work 12 h shifts, whereas others have more traditional 8 h work days, with part time hours available. Staff numbers range from 33 to 2000, and consist of a range of age groups, and varying gender ratios. Some have a budget for well-being activities, and provide individual benefits, whilst others do not. Some centres already had staff well-being initiatives in place prior to SUH. This range of factors leads to significant variations in workplace culture between the study sites. This means that we expect to achieve greater statistical power in a stepped wedge design compared to standard designs, since stepped wedge designs make greater utilisation of within-site comparisons and multiple measurements within sites [46]. We do note however the “incomplete” nature of measurements in this feasibility study, so that although we do not expect much gain in statistical power in the current study, we would for a future stepped wedge trial that has more complete measurements.

In summary, by having a stepped wedge design, this enables us to achieve a pragmatic design that properly takes into account the study context and what is acceptable from the perspective of all stakeholders, including contact centres and employees.

The above reasons for the design are similar to those given in favour of the stepped wedge design for the PRIME study [53]. Such designs are increasingly popular, [32] and the results from such studies have been published in high impact journals (e.g. Shah et al. [55] and Norman et al. [54]).

Since this is a feasibility study, the main aim of the trial is to assess feasibility outcomes rather than conduct confirmatory statistical analysis. We are primarily interested in the *feasibility* of randomising sites to sequences, implementation of the intervention condition and collecting data across sites in different cluster-periods. This is our main justification for an “incomplete measurement” approach, which would reduce the resource cost and burden in the trial, and yet useful feasibility data can still be collected.

Secondarily, the “open cohort” design means that the workforce under evaluation is expected to remain mostly the same across the study duration and so the cost of leaving out the measurement of some cluster-periods is not expected to be as high as in continuous recruitment designs, whereby there are different set of participants in each cluster period. For example, in the HighSteacs trial (a continuous recruitment design) [55], if participants were only recruited in some cluster-periods, then this would reduce the sample size of participants dramatically, whereas this would not happen in open cohort designs.

### Sample size

In the SUH intervention, all employees at a site are likely to be exposed to some or all of the intervention activities, or at least be aware of these. However, employees will have the option of taking part in the research evaluation component. Therefore, when we discuss the sample size, we are referring to the number of people taking part in the evaluation (not those only taking part in intervention activities). The sample size and target difference are the same as another similar study using a standard parallel-group cluster randomised design that proposed a sample size of 160 per arm to detect a reduction in workplace sedentary behaviour of 45 min per day [56]. Considering the stepped wedge design for Stand Up for Health, the focus is on the number of participants per cluster period rather than per trial arm, and Fig. 1 shows that we have at least 6 vertical cross sectional comparisons between control and post-intervention. Therefore in order to get 160 participants included in the vertical comparisons, we need at least 160/6 = ~ 27 individuals per contact centre per data collection period. We initially planned to recruit 10 contact centres although 11 contact centres expressed interest and met the criteria for inclusion. This number of sites was considered large enough to ensure a diverse range of contact centres and enable us to gather preliminary estimates of the between-site variability. A total sample size of 297 participants is therefore expected. One of the aims of this feasibility study is to test sample size assumptions and produce a more accurate sample size calculation for a future study.

### Inclusion criteria

All staff of working age (16 years or older) in the participating contact centres had (or will have) the opportunity to take part; however, some additional criteria were necessary to ensure appropriate collection of data as per the study design:

1. Provisionally scheduled to work during the 7 days of data collection of the primary behavioural outcome measure (activPAL activity monitor), aside from scheduled non-work days.

2. Not planning to leave the company in the 3 months after recruitment.

### Randomisation

DS and JM compiled a complete list of the contact centres who agreed to take part in the study; the list was numbered 1 to 11, and then was signed and dated. Randomisation of contact centres to sequences was conducted in May 2019, using computer-generated block randomisation of time points (T1, T2, T3, T4, T5) to sites, stratified by centre size (≤ 500 employees versus > 500 employees). Stratification by centre size ensured that we did not by chance have a large imbalance in the combined centre size across time points. Randomisation was conducted by RP, who was fully blinded to the names of the contact centres, and who generated a list of centre numbers showing the sequences that each centre should be allocated to (first column in Fig. 1).

### Recruitment

Recruitment will be conducted by DS and JM mainly at the control condition data collection time point in Fig. 1, but recruitment will also be allowed at follow-up time points in Fig. 1. Informed consent will be taken prior to data collection, after participants have read through the information sheet and had the opportunity to ask questions of the researchers.

The intervention will be delivered at the centre level, so all employees will have the opportunity to experience the intervention. However, employees have the choice as to whether they will take part in the evaluation. It is therefore plausible that those employees who are the most enthusiastic about the intervention, and potentially motivated to change their behaviour will be recruited and so those taking part may not be entirely representative of all employees at the call centre. However, we would expect that those recruited are also those who engage most strongly with the intervention, so that if there really is a benefit from using the intervention then we will be more likely to identify it in this study.

### Methods to minimise bias

Randomisation of the centres to time points will (in theory) allow us to introduce the intervention to each site in an unbiased way unrelated to time factors or the particular circumstances of each site. It also helps to ensure there is an approximate balance on average across all the intervention start times in terms of participant or contact centre characteristics. Dates when the sites actually started the intervention will be recorded (if different from the planned start times).

Randomisation was conducted independently of the team recruiting centres and so this rules out selection bias at the centre level. Nevertheless, due to the nature of the intervention, participants and the personnel recruiting participants cannot be blinded. Therefore, selection bias at the participant level is a possibility whereby knowledge of the allocation condition will determine who is recruited. However, this possibility is mitigated for two key reasons. Firstly, the randomisation list will be kept hidden from all contact centres and study investigators except DS and JM who will contact sites to arrange a suitable time to introduce the intervention. Where possible, allocation concealment will be used whereby centres will only be informed about exactly when they will start the intervention when they have to know in order to arrange time to organise the intervention visit. Secondly, the open cohort design means that we would expect a significant overlap in the participants recording data under the control and intervention conditions. We will make every effort to follow-up participants recruited under the control condition and collect their follow-up data to minimise the possibility of differential selection bias between intervention and control conditions and/or attrition bias. Moreover, a sensitivity analysis on the primary outcome will involve including only those participants recording data at multiple time points within each cluster.

Respondent bias is also a possibility due to the lack of blinding, whereby participants may be more likely to change their responses to the questionnaires according to whether their centre is under the intervention or control conditions, and these responses may be disconnected from reality. However, we will also include objective measurements based on the activPAL device that is anticipated to be less affected by biases.

It is possible that staff may move between contact centres allocated to different trial arms, introducing the potential for contamination, however, we expect that this number will be very small (if any) and will have little impact. In any case, we have included a question in the baseline questionnaire asking if participants have previously worked for a company using the Stand Up for Health intervention. We will also attempt to ask participants when they leave a centre if they intend to work at another centre included in the study.

A statistician who is blinded to the names of contact centres and participants will perform the statistical analysis.

### Study outcomes

Study outcomes will be measured at all of the data collection time points (“D”) indicated in Fig. 1. As this is a feasibility study, we will be testing out the methods of collecting data on outcomes, as well as preliminary estimates of effectiveness. The primary outcome is objectively measured sedentary time in the workplace, measured over seven continuous days, using an activPAL™ device.

Secondary outcomes include the following:

1. Subjectively measured sedentary time in the workplace, as measured by The Occupational Sitting and Physical Activity Questionnaire (OSPAQ) [57]
2. Objectively measured (activPAL™ device) prolonged sitting time in the workplace (bouts of ≥ 30 min)
3. Objectively measured (activPAL™ device) total sedentary time (i.e. including time outside the workplace such as at home and leisure time)
4. Objectively measured (activPAL™ device) workplace and total standing time
5. Objectively measured (activPAL™ device) workplace and total physical activity (based on stepping)
6. Objectively measured (activPAL™ device) workplace and total sit-to-stand transitions
7. Objective measures of productivity, which may include absenteeism, presenteeism, call handling time, time spent talking, time spent on hold, time spent wrapping up a call, attendance, or sick leave
8. Utrecht Work Engagement Scale (UWES) [58]
9. Mental well-being as measured by the Warwick-Edinburgh Mental Well-being scale (WEMWBS) [59]
10. Musculoskeletal health as measured by the MSK-HQ [60]
11. Scottish Physical Activity Screening Question (Scot-PASQ) [61]
12. Activities questionnaires to measure the use of activities and preference
13. Staff turnover: number of people leaving and number of new joiners over the study period

### Progression criteria

In this feasibility study, we have set pre-specified progression criteria to determine whether we can proceed to a larger future study. We will apply for further funding if all of the following criteria are satisfied:

1. A 95% confidence interval for the primary outcome includes a clinically relevant reduction in sedentary time of 45 min per day or greater in favour of the intervention. This would reflect substantial progression toward accumulating recommended quantity of 2 h/day standing/light activity during working hours for employees in predominantly desk-based occupations. Given that the sample size calculation is based on a previous study [56] specifying 90% power to detect a 45-min difference, presumably at the 5% significance level, then we would expect that if the observed difference is zero or in favour of control then our 95% confidence interval will not include the 45-min difference. This is especially true given our final sample size is expected to be much greater than the 160 sample size target of the previous study (in the region of 297).
2. The intervention was successfully delivered in at least five of the sites within the study period, and if at least one person in each site was able to use/experience at least one activity.
3. Primary and secondary outcome data was collected in at least 75% of participants overall.
4. Contamination between sites is low or else it is envisaged that contamination can be addressed in the study design of a future study.
5. It is envisaged that any practical difficulties in delivering the intervention across multiple sites or in measuring effectiveness can be overcome when conducting a future large-scale study.

### Statistical analysis plan

#### Assessment of recruitment rate and loss to follow-up

Our focus in the statistical analysis will be mainly on assessing feasibility, including examination of the recruitment rate and loss to follow-up in the study. Information on the timing and reason for any participant withdrawals or loss to follow-up will be collected where possible. We will calculate the following proportions:

(i) The proportion of participants who consent to take part in the evaluation out of the total number of employees (overall and in each centre), with 95% confidence intervals.
(ii) The proportion of participants who drop-out or who are lost-to-follow-up out of the total number of employees (overall and in each centre), with 95% confidence intervals.
(iii) Related to the above, we will record the proportion of participants who leave the company in between consent and 6/9 months follow-up (overall and in each centre), with 95% confidence intervals.
(iv) The proportion of participants who state that they have previously worked for a company which has used the Stand Up for Health intervention.

#### Baseline data analysis

Centre-level descriptive information will be given for the contact centres, including centre size, location, shift patterns, gender split, and age range of workforce. Participant-level descriptive statistics will be computed based on data collected at baseline: including age, gender, employment type, and length of time working in the centre. Categorical baseline data will be presented using counts and percentages, whilst continuous variables will be presented using the mean, median, standard deviation (SD), minimum, maximum, first quartile, third quartile, and number of participants with a response (*n*). Baseline data will be presented overall and stratified by (i) site, and (ii) by allocated sequence group (i.e. T1, T2, T3, T4, T5).

#### Descriptive analysis of outcome data

All study outcomes will be summarised descriptively, split by intervention condition. Additionally, we will summarise the primary outcome split by both site and by intervention condition. Outcome data will be presented in a similar format to the baseline data: counts and percentages for categorical variables, whilst continuous variables will be presented using the mean, median, standard deviation (SD), minimum, maximum, first quartile, third quartile, and number of participants with a response (*n*). We will also summarise the number and proportion of missing data for each outcome.

#### Assessing the feasibility of the planned statistical models for the main trial

In a future larger multi-site study, it is our intention to fit the following statistical model:

A linear mixed effects regression model will be fitted to our primary outcome of objectively measured sedentary time in the workplace, with participants nested within site (both random effects), adjusting for calendar time since the start of study (as a categorical variable in months), and including two dummy variables for whether the intervention has been implemented for 6 or 9 months respectively (i.e. whether data was collected in the first or second follow-up time points).

Other continuous secondary outcomes (including subjectively measured sedentary time in the workplace, prolonged sitting in the workplace, total sedentary time, workplace standing time, total standing time, workplace and total sit-to-stand transitions, objectively measured workplace and total physical activity, subjective physical activity, mental well-being, and musculoskeletal health), will be analysed using the same linear mixed effects regression analysis as above.

We will perform the same analysis in this feasibility study for four reasons as follows:

(i) To assess the feasibility of applying this methodology in practice, especially with respect to assessing model fit/convergence and the magnitude of the standard errors.
(ii) To calculate 95% confidence intervals (CI) for the intervention effect sizes to provide a guide to indicate the likely true effect sizes in the population, and the likely effect sizes that will be observed in a future study. For the primary outcome, this 95% CI will be used to assess if the first progression criterion is met: namely, that our 95% CI includes a 45 min per day reduction in sedentary time.
(iii) To provide preliminary estimates of the between-contact centre variability and within-contact centre variability based on variances produced in the model output, as a means to assess the heterogeneity of quantitative outcomes across sites.
(iv) To explore the effects of missing data and staff turnover using sensitivity analyses to aid the design of a definitive trial. This will involve imputing plausible, but extreme, values for any missing data (e.g. highest recorded sedentary time), whilst also using any information available on the reasons for missing data (if available).
(v) To explore the effect of excluding participants only recording data at a single time point, to assess if this type of sensitivity analysis is possible in the definitive trial, and whether it has an impact on the results. If results change substantially then this may be due to possible selection bias across time points.

The linear mixed effect model results in this feasibility study will be interpreted as exploratory rather than confirmatory. This means that we will acknowledge the need for future studies to confirm our findings.

Linear mixed effects models will be based on the actual times the sites started the intervention rather than the “as-randomised” times, mainly because this is a feasibility study and we wish to reliably assess efficacy with respect to the first progression criteria. However, for the primary outcome analysis, we will also separately fit a model based on the “as-randomised” times to investigate if this makes a difference.

#### Analysis of staff turnover

We will investigate if some types of participants are more likely to leave the company due to staff turnover than others, which will inform the design of the future study. This will involve fitting a logistic mixed regression model to a binary outcome variable indicating whether or not employees who initially consent to the study evaluation have left the company within 6 months (yes or no), adjusting for site as a random effect, and including the following covariates: age, gender, employment type, and length of time working in the centre. If the proportion leaving the company is very low and the model does not converge, we will consider other analysis methods (e.g. univariate analyses). We will repeat the same analysis considering 9 months follow-up, and for loss to follow-up in general.

#### General analysis principles

Analyses will include all sites who agreed to take part in SUH regardless of the circumstances of implementation of the intervention or subsequent site withdrawal. Any sites for which the intervention was not implemented at the correct time or failed to be fully implemented will still be included provided that outcome data are available on participants. Similarly, we will include all eligible participants in the analysis.

There will be no interim analysis of the trial data and the statistical analysis will be performed at the end of the study.

### Health economics

The health economic component aims to lay the groundwork for any future definitive trial, from (a) an NHS and PSS perspective (following the NICE reference case [62] to maximise UK policy relevance), and (b) an employer’s perspective (which may be important at an implementation stage to leverage commercial support).

With regards to the NHS and PSS perspective, whilst it is possible that fitness level improvement may be observable within a trial, it is not anticipated that such fitness would be likely to translate into changes in “hard” health outcomes or patterns of healthcare utilisation (i.e. primary or secondary care utilisation) within an observable trial period. Hence, standard “within trial” style analyses are not anticipated to be appropriate. Instead, the health economic component of the study will focus on scoping the possibility for future economic modelling of longer-term outcomes through consultations with managers and clinicians regarding possible model structures and associated data sources, with targeted literature searches for potential parameter sources such as cardiovascular risk equations. These will be compiled into a short report documenting possible modelling inputs or data gaps with a qualitative assessment of how feasible such modelling would be and the anticipated usefulness of any simulated outcomes to an NHS and PSS decision-maker.

With regards to an employer’s perspective, within-trial observations will be limited to estimates of the cost of direct implementation costs, such as workstation adjustments, SUH equipment, information sessions or similar presented alongside descriptive statistics regarding the rates of installation of each, and measures of productivity and absenteeism. With regards to absenteeism and productivity measures, consultations will be held with management staff to ascertain what measures they would find most useful and are potentially available to collate. Whilst specific details are to be determined it is anticipated that these will be in natural units such as total attendance/sick leave, call handling time, time spent on hold, time spent wrapping up a call, or similar metrics used internally by the centres to measure performance. It is also anticipated that many of these may be available in aggregate level only. Outcomes of interest will be documented; availability of data and suitability of data for use in a future trial will be reported. Where data is available, descriptive metrics and rates of missingness will be reported as deemed appropriate with discussion around their likely benefit and practicality for use in future trials.

## Discussion

Stepped wedge studies are a pragmatic and cost-effective way of undertaking feasibility studies for public health interventions. They enable both the intervention and the study procedures (e.g. recruitment and data collection) to be modified and developed over time in a way that parallel-group designs do not facilitate. This results in greater learning about what is feasible and gathering of information on contextual factors including the size of individual centres, their function and purpose; their environmental and their cultural constraints. Information gathered from this feasibility study will help determine the feasibility of conducting a future multi-site study using a stepped wedge design with a larger number of contact centres and inform the power calculation for a future trial. It will enable both the intervention and the study procedures to be adaptive and responsive to the complex system in which they are being delivered and implemented. Without taking such an approach, both the effectiveness of the intervention and any evaluation design are likely to be sub-optimal.

Nevertheless, there are some particular challenges associated with stepped wedge designs [63]. Of particular relevance to our study are the challenges with maintaining centre-level and participant-level engagement, challenges with ensuring the intervention start times follow the pre-planned schedule [63], increased risk of drop-out or contamination due to the longer study duration, and the possibility of residual confounding due to natural secular changes over time.

A large part of the intervention depends on the level of support, enthusiasm, co-operation and co-ordination of the participating contact centres, and the key staff with whom the researchers liaise with. This heavily impacts on the context and manner with which the intervention is delivered, and how it is received by staff and management. More importantly, the amount of effort put forth by the centre to recruit, what tactics are used and how it is framed to staff, impacts on participant numbers. The researchers can only control and guide so much of this through correspondence. One study assigned a dedicated staff member, who worked for and was paid by the organisation as the research liaison, assisting with recruitment and intervention logistics [56]. This may have been helpful in the current study by improving engagement and organisational buy-in within the centres. This study will be supplemented by a qualitative component to explore these aspects, and the acceptability of data collection methods in greater detail.

To our knowledge, this is the first stepped wedge feasibility study conducted in call centres. The rationale and justification of our novel staircase stepped wedge design has been presented, and we hope that by presenting our study design and statistical analysis plan it will contribute to the literature on stepped wedge trials, and in particular feasibility stepped wedge trials. The findings of the study will also help inform whether this is a suitable design for other settings where data collection is challenging.

## Data Availability

No datasets were generated or analysed to produce this article. After the study is complete, any requests for data sharing should be directed to ECTUdatashare@ed.ac.uk
